# COVID-19 control strategies in Taizhou city, China

**DOI:** 10.2471/BLT.20.255778

**Published:** 2020-07-08

**Authors:** Haijiang Lin, Congcong Guo, Yafei Hu, Hongbiao Liang, Weiwei Shen, Wenhui Mao, Na He

**Affiliations:** a Taizhou City Center for Disease Control and Prevention, Zhejiang Province, China.; b Jiaojiang Center for Disease Control and Prevention, Taizhou of Zhejiang Province, China.; c Duke Global Health Institute, Duke University, Durham, United States of America.; d Department of Epidemiology, School of Public Health, Fudan University, 138 Yixueyuan Road, Shanghai 200032, China.

## Abstract

**Problem:**

On 21 January 2020, the city of Taizhou, China, reported its first imported coronavirus disease 2019 (COVID-19) case and subsequently the number of cases rapidly increased.

**Approach:**

To organize the emergency responses, the government of Taizhou established on 23 January 2020 novel headquarters for prevention and control of the COVID-19 outbreak, by coordinating different governmental agencies. People at high risk of acquiring COVID-19, as well as probable and confirmed cases, were identified and quarantined. The government closed public venues and limited gatherings. The Taizhou Health Commission shared information about identified COVID-19 patients and probable cases with affected agencies. To timely track and manage close contacts of confirmed cases, Taizhou Center for Disease Control and Prevention did epidemiological investigations. Medical institutions or local centers for disease control and prevention reported confirmed cases to the national Center for Disease Control and Prevention.

**Local setting:**

Taizhou, a city in Zhejiang province with about 6 million residents, reported 18 confirmed COVID-2019 cases by 23 January 2020, which ranked it third globally in number of cases after Wuhan and Xiaogan cities in the Hubei province.

**Relevant changes:**

In total, 146 confirmed cases (85 cases imported and 61 cases through community transmission) and no deaths due to COVID-19 had been reported in Taizhou by 1 June 2020. Between 16 February and 1 June 2020, no confirmed case had been reported.

**Lesson learnt:**

Identifying and managing imported cases and people at risk for infection, timely information sharing, limiting gatherings and ensuring collaborations between different agencies were important in controlling COVID-19.

## Résumé

**Problème:**

Le 21 janvier 2020, la ville de Taizhou, en Chine, a signalé son premier cas importé de maladie à coronavirus 2019 (COVID-19). Le nombre de cas a ensuite rapidement augmenté.

**Approche:**

Afin d'organiser les interventions d'urgence, les autorités de Taizhou ont établi le 23 janvier 2020 un nouveau quartier général chargé de la prévention et du contrôle de l'épidémie de COVID-19, fruit de la coordination entre différents organismes gouvernementaux. Les personnes présentant un risque élevé de contracter la COVID-19, ainsi que les cas probables et confirmés, ont été identifiés et mis en quarantaine. Les autorités ont fermé les lieux publics et limité les rassemblements. La Commission de la santé de Taizhou a partagé des informations relatives aux patients COVID-19 identifiés et aux cas probables avec les agences concernées. Pour retracer et gérer sans délai les contacts rapprochés des cas confirmés, le Centre de prévention et de contrôle des maladies de Taizhou a mené des investigations épidémiologiques. De leur côté, les établissements médicaux ou centres locaux de prévention et de contrôle des maladies ont notifié les cas confirmés au Centre national de prévention et de contrôle des maladies.

**Environnement local:**

Taizhou est une ville de la province du Zhejiang et compte environ 6 millions d'habitants. Elle avait dénombré 18 cas de COVID-2019 confirmés au 23 janvier 2020, ce qui la classait au troisième rang mondial en nombre de cas après Wuhan et Xiaogan, dans la province du Hubei.

**Changements significatifs:**

Taizhou a enregistré un total de 146 cas confirmés (85 cas importés et 61 cas de transmission communautaire) et aucun décès dû à la COVID-19 au 1^er^ juin 2020. Entre le 16 février et le 1^er^ juin 2020, aucun cas confirmé n'a été signalé.

**Leçons tirées:**

Identifier et gérer les cas importés ainsi que les personnes à haut risque d'infection, partager les informations au moment opportun, limiter les rassemblements et assurer une bonne collaboration entre les différents organismes sont autant de mesures qui jouent un rôle essentiel dans le contrôle de la COVID-19.

## Resumen

**Situación:**

El 21 de enero de 2020, la ciudad de Taizhou (China) comunicó su primer caso importado de la enfermedad del coronavirus 2019 (COVID-19) y posteriormente el número de casos se incrementó rápidamente.

**Enfoque:**

El 23 de enero de 2020, el gobierno de Taizhou estableció una nueva sede para la prevención y el control del brote de la COVID-19, a través de la coordinación de distintos organismos gubernamentales para organizar las respuestas de emergencia. Se identificaron y entraron en cuarentena las personas con alto riesgo de padecer la COVID-19, así como los casos probables y confirmados. El gobierno cerró los lugares públicos y restringió las reuniones. La Comisión de Salud de Taizhou compartió información sobre los pacientes identificados con la COVID-19 y los casos probables con los organismos afectados. El Centro para el Control y la Prevención de Enfermedades de Taizhou realizó investigaciones epidemiológicas para hacer un seguimiento oportuno y manejar los contactos cercanos de los casos confirmados. Las instituciones médicas o los centros locales para el control y la prevención de enfermedades reportaron los casos confirmados al Centro Nacional para el Control y la Prevención de Enfermedades.

**Marco regional:**

Taizhou, una ciudad de la provincia de Zhejiang que tiene unos 6 millones de habitantes, comunicó 18 casos confirmados de la COVID-2019 al 23 de enero de 2020, lo que la situó en el tercer lugar mundial en número de casos después de las ciudades de Wuhan y Xiaogan en la provincia de Hubei.

**Cambios importantes:**

En total, para el 1.º de junio de 2020 se confirmaron 146 casos (85 casos importados y 61 casos por transmisión comunitaria) y no se notificaron muertes por causa de la COVID-19 en Taizhou. Tampoco se notificaron casos confirmados entre el 16 de febrero y el 1.º de junio de 2020.

**Lecciones aprendidas:**

La identificación y la gestión de los casos importados y de las personas en riesgo de infección, el intercambio oportuno de información, la restricción de las reuniones y la garantía de la colaboración entre los distintos organismos fueron importantes para controlar la COVID-19.

## ملخص

المشكلة في 21 يناير/كانون ثاني 2020، أعلنت مدينة تايتشو في الصين، عن أول حالة واردة للإصابة بفيروس كورونا 2019 (كوفيد-19)، وبالتالي ازداد عدد الحالات بسرعة.

الأسلوب بغرض تنظيم الاستجابات في حالات الطوارئ، قامت الحكومة في تايتشو في 23 يناير/كانون ثاني عام 2020 بإنشاء مقر جديد للوقاية من تفشي كوفيد-19 والسيطرة عليه، عن طريق تنسيق الوكالات الحكومية المختلفة. تم تحديد الأشخاص المعرضين لخطر كبير للإصابة بفيروس كوفيد-19، وكذلك الحالات المحتملة والمؤكدة، وإخضاعهم للحجر الصحي. أغلقت الحكومة الحدائق العامة ومنعت التجمعات المحدودة. قدمت لجنة الصحة في تايتشو معلومات حول مرضى كوفيد-19 الذين تم تحديدهم، وكذلك الحالات المحتملة، مع الوكالات المعنية. ولتتبع المخالطين عن قرب مع الحالات المؤكدة، والتعامل معهم في الوقت المناسب، قام مركز تايتشو لمكافحة الأمراض والوقاية، بإجراء تحقيقات وبائية. قامت المؤسسات الطبية أو المراكز المحلية لمكافحة الأمراض والوقاية منها، بالإبلاغ عن الحالات المؤكدة للمركز الوطني لمكافحة الأمراض والوقاية منها.

المواقع المحلية أعلنت مدينة تايتشو في مقاطعة تشيجيانج، والتي يبلغ عدد سكانها حوالي 6 ملايين نسمة، عن 18 حالة مؤكدة للإصابة بكوفيد-19 بحلول يوم 23 يناير/كانون ثاني 2020، مما جعلها تأتي في المرتبة الثالثة على مستوى العالم من حيث عدد الحالات بعد مدينتي ووهان وتشياوجان في مقاطعة هوبي.

التغيّرات ذات الصلة في المجمل، تم الإبلاغ عن 146 حالة مؤكدة (85 حالة واردة، و61 حالة عن طريق الانتشار في المجتمع)، ولم يتم الإبلاغ عن أي حالة وفاة بسبب كوفيد-19 في تايتشو بحلول 1 يونيو/حزيران 2020. وخلال الفترة ما بين 16 فبراير/شباط و1 يونيو/حزيران 2020، لم يتم الإبلاغ عن أية حالة مؤكدة.

الدروس المستفادة كان من المهم للسيطرة على كوفيد-19، تحديد وإدارة الحالات الواردة والأشخاص المعرضين لخطر الإصابة بالعدوى، وتبادل المعلومات في الوقت المناسب، والحد من التجمعات، وضمان التعاون بين الوكالات المختلفة.

## 摘要

**问题:**

2020 年 1 月 21 日，中国台州市报告了首例输入性 2019 冠状病毒病 (COVID-19) 病例，随后病例数迅速增加。

**方法:**

为了组织应急响应工作，2020 年 1 月 23 日，台州政府成立了新型指挥部，以便通过协调不同的政府机构来开展 COVID-19 疫情防控工作。确定并隔离 COVID-19 高风险人群以及疑似和确诊病例。政府关闭公共场所并限制聚会。台州市卫生健康委员会与受影响的机构分享了 COVID-19 确诊患者和疑似病例相关信息。为了及时跟踪和管理确诊病例的密切联系人员，台州市疾病预防控制中心进行了流行病学调查。医疗机构或地方疾病预防控制中心向国家疾病预防控制中心报告了确诊病例。

**当地状况:**

台州市下属浙江省，常住人口约 600 万，截至 2020 年 1 月 23 日，该市已报告 18 例 COVID-2019 确诊病例，在病例数方面排名全球第三，仅次于湖北省的武汉市和孝感市。

**相关变化:**

截至 2020 年 6 月 1 日，台州总计报告 146 例确诊病例（85 例为输入病例，61 例通过社区传播），无 COVID-19 相关死亡病例。2020 年 2 月 16 日至 6 月 1 日期间，无报告的确诊病例。

**经验教训:**

确定和管理输入病例和高风险人群、及时共享信息、限制聚会并确保不同机构之间的合作对于控制 COVID-19 非常重要。

## Резюме

**Проблема:**

21 января 2020 года в городе Тайчжоу, Китай, был зарегистрирован первый ввезенный случай коронавирусной инфекции 2019 (COVID-19), а впоследствии число заболевших стремительно возросло.

**Подход:**

Для организации экстренного реагирования 23 января 2020 года администрация Тайчжоу создала новые штаб-квартиры с целью предупреждения и борьбы со вспышкой COVID-19 посредством координации действий различных правительственных учреждений. Были выявлены и помещены на карантин лица с высокой степенью риска заражения COVID-19, а также лица с подозрением на заболевание и уже подтвержденным заболеванием. Администрация города закрыла общественные места и ограничила общественные собрания. Комиссия по вопросам здравоохранения Тайчжоу поделилась информацией о выявленных пациентах с COVID-19 и вероятных случаях заболевания с затронутыми государственными учреждениями. Для своевременного отслеживания лиц, находившихся в тесном контакте с лицами с подтвержденным заболеванием, и управления ими Центр по контролю и профилактике заболеваний Тайчжоу провел эпидемиологические исследования. Медицинские учреждения или местные центры по контролю и профилактике заболеваний сообщали данные о подтвержденных случаях заболевания в государственный Центр по контролю и профилактике заболеваний.

**Местные условия:**

Тайчжоу, город в провинции Чжэцзян с населением около 6 миллионов человек, к 23 января 2020 года сообщил о 18 подтвержденных случаях заболевания COVID-2019, что позволило ему занять третье место в мире по количеству случаев заболевания после городов Ухань и Сяогань в провинции Хубэй.

**Осуществленные перемены:**

Всего к 1 июня 2020 года в городе Тайчжоу было зарегистрировано 146 подтвержденных случаев заболевания (из которых 85 ввезенных случаев и 61 случай местного контактного заражения), при этом в Тайчжоу не было зарегистрировано ни одного случая смерти от COVID-19. В период с 16 февраля по 1 июня 2020 года не было зарегистрировано ни одного подтвержденного случая.

**Выводы:**

Выявление ввезенных случаев заболевания и лиц, подверженных риску заражения, и управление ими, своевременный обмен информацией, ограничение общественных собраний и обеспечение сотрудничества между различными государственными учреждениями имели особую важность для борьбы с COVID-19.

## Introduction

In December 2019, reports on cases of pneumonia of unknown etiology emerged from Wuhan, China. Subsequently, researchers identified the novel severe acute respiratory syndrome coronavirus 2 (SARS-CoV-2) as the causative agent. The outbreak grew and the virus spread to other cities in China^1^ and across the world. On 11 March 2020, the World Health Organization announced the coronavirus disease 2019 (COVID-19) outbreak a pandemic.^2^^,^^3^

Containing the spread of the virus from imported cases and preventing community transmission is important for controlling virus transmission in the early stage of an outbreak. Here we describe how decision-makers in the city of Taizhou, China, took newly-developed approaches to control the spread of SARS-CoV-2.

## Local setting

In 2018, Taizhou, situated in Zhejiang Province, had about 6 million residents.^4^ The city reported its first COVID-19 case on 21 January 2020. As of 23 January 2020, the city had reported 18 confirmed cases, ranking it the third most affected city after Wuhan and Xiaogan in the Hubei province, China.

On 20 January 2020, the National Health Commission decided that COVID-19 should be classified as a notifiable infectious disease, according to the Chinese law.^5^ Any new confirmed case must be reported to the national Center for Disease Control and Prevention (CDC) within 2 hours through the national reporting system of infectious disease and all health administrations should make the number of daily reported cases publicly available. Furthermore, Chinese citizens must report any issue related to COVID-19 through a government service hotline or contact community workers. For the epidemiological investigation, a citizen must include exposure history, symptoms and close contacts, and anyone who conceals facts could be subjected to criminal detention and other penalties.

## Approach

**Fig. 1:**
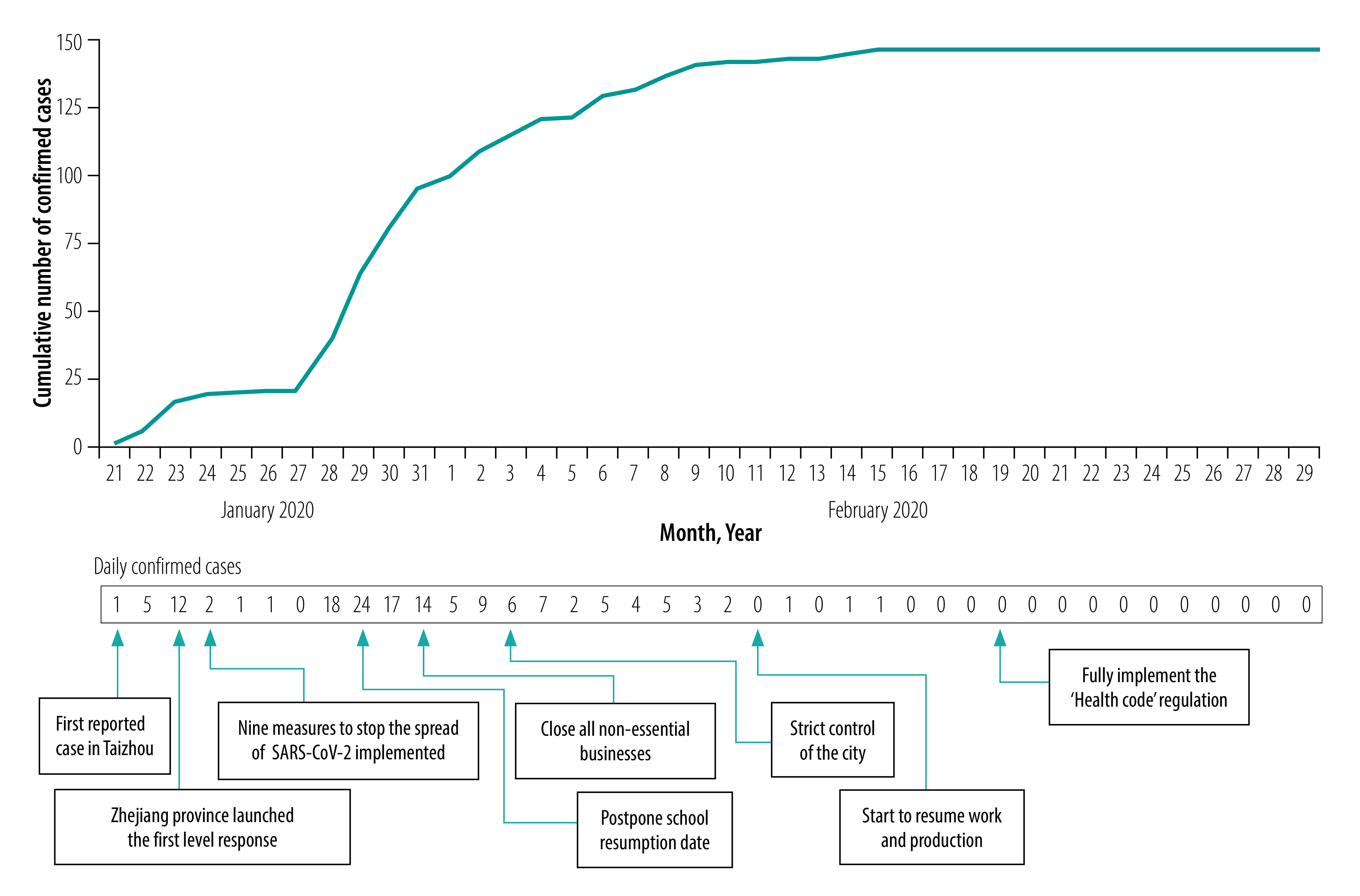
Number of COVID-19 cases and the prevention and control measures taken in Taizhou, China, 2020

To respond to the outbreak, the government of the Zhejiang province launched the first level response to a major public health emergency on 23 January 2020. This launch included the establishment of novel provincial, municipal and district headquarters for COVID-19 epidemic prevention and control, which organized the emergency responses according to the decisions of the Chinese State Council.^6^ The Taizhou municipal headquarters consisted of staff members from the Taizhou Health Commission, Taizhou CDC, hospitals and other affected sectors. On 24 January 2020, Taizhou implemented nine measures to address the increasing COVID-19 cases (Box 1).^7^ Furthermore, school resumption date was announced to be postponed on 29 January 2020 and all non-essential businesses were closed on 31 January (Fig. 1).

Box 1 **Nine measures taken to stop the spread of SARS-CoV-2, 24 January 2020, Taizhou, China**

(i) Cancellation of holidays for main leaders of the Party and governments in counties and towns during the Spring festival (24 January to 2 February 2020);

(ii) medical insurance coverage for all people with confirmed COVID-19 regardless of previous insurance status;

(iii) designated hospitals for COVID-19 care opened and probable patients were given priority treatment;

(iv) medical observation of all close contacts to probable or confirmed cases;

(v) closing of all public gathering places, such as entertainment places, internet cafes, cinemas, libraries and museums;

(vi) cancellation of all public gathering activities;

(vii) people entering hotels, transport stations, airports, and entrances and exits of main roads of the community must measure their temperature and report any symptom and exposure history;

(viii) prohibition of live birds and wild animal trades; and

(ix) investigation and, if need, prosecution of people who disrupt order and fabricate rumours.

COVID-19: coronavirus disease 2019; SARS-CoV-2: severe acute respiratory syndrome coronavirus 2.

COVID-19: coronavirus disease 2019.

Note: The schools have re-opened since 13 April 2020.

To manage the high-risk populations efficiently, the Health Commission staff summarized the information of high-risk populations, including their demographic characteristics and exposure history. CDC and medical observation sites shared the information of confirmed cases in real time (within 2 hours from the time when the patient is confirmed) through the national reporting system of infectious disease.

The diagnosis and treatment for COVID-19 were made free of charge, either by basic insurance or government subsidies. All level of governments provided subsidies for medical devices, such as thermometers, respirators, extracorporeal membrane oxygenation systems and negative pressure isolation ambulances.

Headquarters implemented six major control strategies, which we describe below in detail.

### Control strategies

#### Screening and quarantine

To identify people at high risk of acquiring COVID-19, government officials screened people passing traffic entrances to the city, people in the community and people with a fever visiting outpatient clinics. People suspected of having COVID-19 were identified by measuring body temperature and taking travel and/or contact history. Identified high-risk people were required to stay at home for 14 days. The isolated person had to measure his or her body temperature twice a day, which they reported daily via phone to the health-care workers or community workers. Health-care workers could also make home visits to measure temperature. Those who had a temperature of 37.3 °C or higher, judged by a health community worker, were sent to designated medical institutions.

Since 22 January 2020, people who had visited the Hubei provinces were regarded as a high-risk population and since 29 January 2020, the same criteria applied for people who had visited Wenzhou city, Zhejiang province. To identify this population, the Taizhou´s governmental big data bureau analysed data of mobile phone movement. Community health workers identified people in their homes by door-to-door visits.

At headquarters, a special taskforce, composed by personnel from the big data bureau, public security bureau and the health commission of Taizhou, collected and managed the data on the demographic characteristics and exposure history of this population. Before the Taizhou electronic epidemic prevention and control information management system was established, they managed the data by using paper-based records.

#### Traffic entrance management

On 24 January 2020, the public security bureau established checkpoints to inspect passengers entering Taizhou through highways, railways and airplanes. Each community or village had only one entrance open, where a checkpoint was established. Each checkpoint was staffed with at least one worker from the public security department and one worker from the local primary health centre. The checkpoint staff inquired anyone presenting with respiratory symptoms and/or a temperature of 37.3 °C or higher (when measured by a hand-held thermometer) about their travel, residence and close contact history in the past two weeks.

Depending on the risk, the checkpoint staff used different observation and quarantine strategies. For people with exposure history and fever or respiratory symptoms, checkpoint staff sent paper-based information regarding demographic characteristics and exposure history of the identified person to headquarters. An ambulance took the patient to the local fever clinic in the designated hospitals.

If there were accompanying individuals in the car, they were required to be home quarantined. People with exposure history, but no symptoms in the checkpoints were sent to the medical observation site, which was a converted hotel now containing a medical examination room, a medical observation area, an office and living area for staff members and a logistic support area. People without exposure history, but with a fever were sent to the local fever clinic to exclude the possibility of having COVID-19, by using SARS-CoV-2 nucleic acid test, chest X-ray and blood tests. People testing negative were followed up by community health workers through a telephone call or home visit. People without exposure history or fever could pass through the checkpoint.

#### Medical observation site

The medical observation site is composed of at least one public security staff, one clinician and one nurse. The nurse measured the person’s body temperature twice a day and asked for the symptoms, especially cough and shortness of breath. If the person presented symptoms, s/he would be sent to a designated COVID-19 hospital. After the 14-day observation period the person would be released if no symptoms had been shown.

#### Medical institutions

There were 27 designated hospitals for the diagnosis and treatment of COVID-19 across the nine counties in Taizhou. Personnel wore work clothes, disposable work caps, disposable gloves, disposable medical protective clothing, medical protective masks or powered air supply filter respirators, protective screens or goggles and work shoes or rubber boots with waterproof boot covers. In the reception, a senior nurse would identify probable cases based on people’s body temperature, respiratory symptoms and exposure history. A specialized expert group from the infection department, respiratory department and radiation department would jointly provide consultation for the patients. Confirmation of COVID-19 was done with a SARS-CoV-2 nucleic acid detection kit (Shanghai BioGerm Medical Biotechnology Co., Ltd, Shanghai, China).

#### Contact tracing

For each confirmed or probable case, local CDC professionals did an epidemiological investigation within 24 hours of diagnosis by consulting data and by interviewing to understand the natural history of each case and to trace the source of infection and close contacts. Anyone who has had a close contact (that is, within 1 m distance) within two days before the onset of any symptoms in the confirmed patient was regarded as a close contact. On aircrafts equipped with air filters, passengers who were sitting up to three rows in front of or behind the patient, plus the aircrew, were regarded as close contacts. On trains, buses, ferries or aircrafts without air filters, all passengers were regarded as close contacts.

Close contacts were sent to the local medical observation site for a 14-day quarantine and were tested for SARS-CoV-2 when symptoms appeared. If they were confirmed to be having COVID-19, their residences were disinfected by CDC professionals.

#### The Health Code

Since 19 February, the government of Taizhou has been using the Health Code, a quick response (QR) code showing the probability of a person having COVID-19. All citizens had to download the mobile application, such as Alipay or WeChat, containing the QR code to their smartphones. They filled in a questionnaire about their symptoms and exposure history in the applications when applying the QR code for the first time. When entering into traffic checkpoints, workplaces, communities and other public places they had to display the real-time Health Code and their temperatures were measured. The Health Code will be marked as red for confirmed or probable patients and they would be sent to the designated hospital immediately. Other high-risk populations, such as people from cities with a severe epidemic and contacts of confirmed or probable patients would also be marked with a red code and require a 14-day home quarantine. People with fever or any respiratory symptoms, but without other risk factors, such as exposure to high risk areas, would be marked with a yellow code and require a 14-day home quarantine. After filling in the personal health information in the smartphone application every day during the quarantine period, the red code would automatically turn green at the end of the quarantine if the person was symptom free. If a person entered symptoms into the Health Code during quarantine, s/he would be sent to a designated COVID-19 hospital. Residents without any symptoms or risk factors would be marked with a green code and could pass through all checkpoints freely.

## Relevant changes

Fig. 1 shows the timeline of events and the number of confirmed cases per day. After the outbreak reached its daily peak of new cases on 29 January 2020, the epidemic situation rapidly improved. On 11 February 2020, no new cases were reported for the first time and by 11 March 2020 all hospitalized patients had been discharged from the hospitals. Up to 1 June 2020, 146 confirmed cases (85 cases imported and 61 cases through community transmission) and no deaths due to COVID-19 had been reported. As of 1 June 2020, all townships were deemed at the lowest risk for an outbreak.

In total, 85 796 people had been quarantined by 29 February 2020. Of those, 30 813 people from Hubei and 40 614 people from Wenzhou had been traced in Taizhou. The ratio of secondary cases to imported cases was 0.72: 1, indicating that the control strategies had effectively eased the transmission from imported epidemic to the local community.

## Lessons learnt

Prevention of community-level transmission from imported cases was an effective strategy in containing the spread of COVID-19 in Taizhou. Through active screening strategy among the high-risk populations to detect early-stage cases and effective control strategy on infected patients, probable cases and their close contacts, the government of Taizhou minimized the risk of transmission. However, the prevention and control work had some challenges. First, the disease prevention and control system lacked capacity for a major public health emergency and personal protective equipment needed for the work was insufficient. Therefore, the government integrated the resources of various agencies and organizations, created fever clinics in medical institutions, strengthened the monitoring and early warning of public health events and purchased personal protective equipment. The government also supported manufacturing enterprises by reducing taxes and giving preferential loans to incite production of medical supplies and personal protective equipment. Second, finding all high-risk people and COVID-19 positive individuals was cumbersome. Hence the government ensured that the information was shared among different departments, and big data analytics and community screening were used, as well citizens mobilized for reporting any issue related to COVID-19. Third, to address the panic felt by some citizens, the government strengthened health education and publicity to citizens, shared information and acted in a timely manner to the epidemic.

Several lessons could be learnt from this experience (Box 2). First, identifying and managing high-risk populations based on symptoms and exposure history require accommodating the changes in the outbreak, such as multiple epicentres. Second, timely sharing of information and epidemiological investigations are helpful to track and manage close contacts of confirmed cases. Third, timely closure of public venues and limiting public gatherings reduce the risk of infection in the general population. Fourth, joint efforts across different government agencies are needed for an effective response. Finally, strict implementation of the control measures ensures that probable cases are rapidly detected, which reduces the spread of SARS-CoV-2.

Box 2 **Summary of main lessons learnt**

- Quick responses and joint efforts across different government agencies are of great importance in controlling the emerging infectious disease.
- Detecting cases and identifying high-risk populations timely requires accommodating the changes in risk factors.
- Timely closure of public venues and limiting gatherings are crucial for reducing the transmission of infectious diseases.
